# Editorial: Humor and Laughter, Playfulness and Cheerfulness: Upsides and Downsides to a Life of Lightness

**DOI:** 10.3389/fpsyg.2019.00730

**Published:** 2019-04-09

**Authors:** Willibald Ruch, Tracey Platt, René T. Proyer, Hsueh-Chih Chen

**Affiliations:** ^1^Department of Psychology, University of Zurich, Zurich, Switzerland; ^2^Department of Psychology, University of Sunderland, Sunderland, United Kingdom; ^3^Department of Psychology, Martin-Luther-University Halle-Wittenberg, Saxony-Anhalt, Germany; ^4^Department of Psychology, National Taiwan Normal University, Taipei, Taiwan

**Keywords:** humor, playfulness, laughter, cheerfulness, gelotophobia, wit

## Introduction

This research topic brings together the four research areas of humor, laughter, playfulness, and cheerfulness. There are partial overlaps among these phenomena. Humor may lead to laughter but not all laughter is related to humor. Playfulness is considered the basis of humor (a play with ideas), but not all play is humorous. Cheerfulness is considered the temperamental basis of good humor, a disposition for laughter and for keeping humor in face of adversity but it mostly overlaps with the socio-affective component of humor. Laughter was considered a play signal and to indicate the annulment of seriousness, but there is play without laughter and laughter outside of play. Cheerfulness might facilitate play and cheerful state might be raised due to play but again the conceptual overlap is only partial. They all contribute to levity in life and their apparent similarity suggests studying them together to map out the territory; i.e., to see where they overlap and what is specific. While these traits and behaviors have the potential to contribute to a good life, there is the danger of overlooking their non-virtuous facets; that is, laughter may not only be expressing amusement but scorn directed at people, humor may be benevolent but there is also sarcasm, and playfulness may elicit positive emotions but also risk prone behaviors. While this research topic solicited articles to these four domains without the aim to connect them, a few articles did and it is expected that growing together will be one outcome of this compilation of articles.

Currently, these fields are studied mostly in isolation. A literature search (using the psychology database of Web of Science Core Collection from 1900, 06.08.2018) yielded that humor is clearly leading in terms of number of publications (*n* = 3,006), followed by laughter (*n* = 1,412), playful(ness) (*n* = 629), and cheerful(ness) (*n* = 204). As a comparison, antonyms were studied as well, and yielded higher numbers, such as for crying (*n* = 1640), serious-mindedness (or seriousness) (*n* = 892), and sadness (*n* = 3,654). The latter indicates that sadness is 18 times more frequently researched than cheerfulness.

Next, the frequency of articles combining terms was investigated. Combinations of humor and one of the other key terms are rather infrequent with the exception of “humor and laughter” (*n* = 454), suggesting that about 10% of all articles on humor also refer to laughter. Humor and playfulness (*n* = 59) and humor and cheerfulness (*n* = 53) represent only 2% of all articles on humor, and these numbers are still much higher than any combination among the other three. This clearly shows that work is needed integrating these areas to examine how the concepts overlap both regarding their defining substance but also in predicting third variables. It should be mentioned that in a pioneering publication preceding the renaissance of empirical humor research three of the keywords were considered together. Toronto-based English psychologist (Berlyne, 1969) gave an account of laughter, humor, and play in a chapter in a handbook of social psychology. The compilation of research in the four fields is aimed at deepening our understanding of these concepts and stimulating research combining them.

## Overview of Studies

There are 32 manuscripts in this research topic. Not surprisingly, most articles are on various aspects of humor, followed by laughter (including dispositions to ridicule and being laughed at), playfulness and cheerfulness. To highlight some prevalent issues beforehand: Individual studies relate to introducing new concepts, or new scales or working on existing ones (Aykan and Nalçaci; Bruntsch and Ruch; Heintz et al.; Hofmann et al.; Hofmann et al.; Ruch and Heintz; Ruch et al.; Ruch et al.). Furthermore, substantial attempts are made to develop and evaluate trainings and interventions (Auerbach; Linge-Dahl et al.; Tagalidou et al.; Wellenzohn et al.). There is also a significant number of cross-cultural comparisons (Heintz et al.; Pang and Proyer; Tosun et al.) and systematic literature reviews (Chadwick and Platt; Linge-Dahl et al.). What research questions were posed and what have we learned in the different fields?

### Humor and Humor-Related Traits

Seven contributions relate to humor. Two are systematic reviews summarizing the use of humor in their related fields. Chadwick and Platt's paper draws upon the 32 existing articles on humor with regards to intellectual disability, which they found grouped into eight emergent themes. The paper showed humor to be of importance in social interactions, not only for people with intellectual disabilities but those who support them and highlighted both the positive and the negative role of humor for both groups. However, the authors suggest that future studies should aim for more empirical rigor when investigating this important, yet complex construct. As Heintz et al. highlighted, the terminology of a dichotomized thinking of positive and negative humor may be a too simplistic approach, especially when thinking about fostering positive relationships. For example, employing carers with a propensity for benevolent humor may help forge more than a work relationship, but a friendship.

In the study of humor assessment and interventions in palliative care, Linge-Dahl et al. reviewed 13 papers. The review found that although the papers were difficult to compare, it was clear that humor is an appropriate and useful resource in palliative care of terminally ill patients (in different settings, such as hospices or oncology wards). Given this review accounts for the last 20 years, the authors note that research is still exceptionally limited, although humor interventions showed promising results on many well-being outcomes.

Humor as a quality that can be trained and developed evidently has potential not only to increase well-being in the terminally ill but also to reducing stress, depressiveness, and anxiety in a population of sub-clinical individuals (Tagalidou et al.). This pilot intervention demonstrated encouraging evidence that a humor training can have a stable, long-lasting impact on increasing positive affective states and reducing levels of stress, depressiveness and anxiety. This study also reported a relatively low attrition rate, which would suggest that participants were enjoying themselves, whilst having an overall positive impact on their mental health.

Wellenzohn et al. studied who benefits from online humor-based positive psychology interventions. In Study 1, personality traits were tested and it was the extraverts that benefitted more from the three funny things intervention than introverts did. Remembering emotional events allows reliving the emotion and the extraverts' tendency to positive emotions (i.e., the amusement due to the funny events during the day) apparently contributed to increasing their level of happiness and to lowering their depressive symptoms. In Study 2, no moderating effects were found for sense of humor on the effectiveness of the five humor-based interventions tested. Interestingly, however, changes in sense of humor from pretest to the 1-month follow-up predicted later changes in happiness and depressive symptoms. Thus, increases in sense of humor during and after the intervention are associated with the interventions' effectiveness.

Instruments that measure aspects of humor were investigated in five studies. Heintz et al. investigate responses to the BenCor in 25 samples from 22 countries. The BenCor measures humor aiming at the good and may be seen as a character (as different from personality or temperament) approach to humor. Benevolent humor treats human weaknesses and wrongdoings benevolently, while corrective humor aims at correcting and bettering them. The 12 items exhibited sufficient psychometric qualities in most of the samples. Metric measurement invariance was supported across the 25 samples, and scalar invariance was supported across age and across gender. This study supported the suitability of the 12 marker items of benevolent and corrective humor in different countries, enabling cross-cultural research and eventually applications of humor aiming at the good. Importantly, benevolent and corrective humor were clearly established as two positively related, yet distinct dimensions of virtue-related humor.

Ruch and Heintz study the construct and criterion validity of the HSQ (Martin et al., 2003), which assesses humor styles. They argue that each item entails construct-relevant content (i.e., humor) but also (unwanted) variance produced by the item context. The 32 items were experimentally manipulated to strip off the context or to substitute the humor content by non-humorous alternatives (i.e., only assessing context). Study 1 shows that humor is not the primary source of the variance in three of the HSQ scales with the self-defeating humor style being primarily determined by the context. Study 2 shows that also the relationships of the HSQ with personality were reduced and those with subjective well-being vanished when the non-humorous contexts in the HSQ items were controlled for. For self-defeating, removing the context rendered the results to a positive rather than a negative view of the humor in this humor style. The results suggest that the items of humor instruments warrant careful examination.

Ruch et al. enlarge the list of styles of humor by adding fun, benevolent humor, non-sense, wit, irony, satire, sarcasm, and cynicism and by providing first evidence for the reliability and validity of a set of 48 marker items for their assessment, the Comic Style Markers (CSM). Exploratory and confirmatory factor analyses showed that the eight styles could be distinguished in English- and German-speaking samples, and studying self- and other-reports supported both convergent and discriminant validity. Studies also showed that the scales tapped differentially into personality, intelligence, and character strengths; for example, wit correlated with verbal intelligence, fun with indicators of vitality and extraversion, and while benevolent humor was related to strengths of the heart, the styles related to mock/ridicule (i.e., sarcasm, cynicism, but also irony) correlated negatively with character strengths. The results suggest that more styles may be distinguished than was done hitherto, which is also confirmed by Heintz and Ruch (2019).

Two more studies examine irony in more detail and distinguish between two forms. Bruntsch and Ruch investigate irony in ironic criticisms (i.e., mock positive evaluation of negative circumstances) and ironic praise (i.e., mock negative evaluation of positive circumstances). They introduce the TOVIDA (Test of Verbal Irony Detection Aptitude) containing 26 scenario-based items for the detection of ironic criticism vs. ironic praise. Initial validation is provided by exploring personality and ability correlates of the two TOVIDA scales. Relatedly, Milanowicz et al. study mocking compliments and ironic praise from an interactional gender perspective. The ability to create irony is assessed and related to state and trait anxiety. Male responses were consistently more ironic but both genders used more irony in response to male ironic criticism than to female ironic praise. Anxiety predicted irony comprehension and willingness to use irony. The results enrich the discussion within the framework of linguistic intergroup bias and natural selection strategies.

Also Aykan and Nalçaci introduce a new instrument (ToM-HCAT) for assessment of ToM (i.e., theory of mind) by humor comprehension and appreciation suitable for healthy adult populations. This performance test consisting of cartoons measures perceived funniness, reaction time to perceived funniness decision, and meaning inference. While a first validation is presented (individuals high and low in the Autism Spectrum Quotient differ in the meaning-inference scores of the subscale with the ToM cartoons) it awaits further validation to support the claim it is useful to detect variations in ToM ability in the healthy adult population.

While Heintz et al. study country differences in measured humor traits, Tosun et al. explore lay conceptions of an ideal sense of humor in three countries, namely Iran, United States, and Turkey. As in prior US studies they find that the embodiment of an ideal sense of humor is predominantly a male figure. Country and gender had an impact on relative number of specific humor characteristics. For example, Americans mentioned hostility/sarcasm and caring more often than participants from the other countries. Further work is needed to replicate the observed group differences and to identify their sources.

Canestrari et al. use the Theory of the Pleasures of the Mind to study the enjoyment derived from both humor and insight problem solving as they share similar cognitive mechanisms. The results show that finding the solution to a problem is associated with a positive evaluation, and curiosity, virtuosity and violation of expectations are the most frequent explanations. Understanding a joke is accompanied by the joy of verification and a feeling of surprise. However, the choice for the most enjoyable cartoons related to other factors, such as recognizing a violation of expectations and experiencing a diminishment in the cleverness attributed to the characters in the cartoon.

Mendiburo-Seguel et al. investigate the effects of political humor on an individual's trust toward politics and politicians. They conducted two experiments, in which participants were exposed to political disparagement humor to non-humorous political information, or to non-political humor. Study 1 showed that an exposure to political disparagement humor and non-humorous political contents negatively affects trust in politicians immediately after the exposure. Study 2, in which semidaily messages were sent to the participants, did not yield significant effects.

The study by Wagner nicely demonstrates how close upside and downside of humor are together by showing that class clown behavior was positively related to different indicators of social status and peer-rated popular-leadership behavior, but also to aggressive-disruptive behaviors and negatively to prosocial behaviors. Thus, humor is involved in making a student popular but it may also be used in destructive ways. The study also demonstrates that it is important to distinguish among different dimensions of class clown behavior, as they yielded different results.

### Laughter and Dispositions to Ridicule and Being Laughed at

Laughter is both a social signal and an expression of emotion with several behavioral and physiological components (e.g., respiratory, acoustic, facial, postural, hormonal). There are different motivations for laughter (with laughing *with* and laughing *at* being a minimal distinction made by many) and there are individual differences to be considered regarding both the laughing person and the one perceiving the laughter. Laughter is studied among the healthy but also within psychopathology. Clearly, the section of this research topic devoted to laughter and laughter-related dispositions received a variety of submissions.

Ritter and Sauter investigated whether listeners can identify in- and out-group members from laughter. They showed that listeners were unable to accurately identify group identity from laughter and the exposure to a group did not affect the classification performance. In conclusion, group membership cannot be inferred from the way people laugh.

Curran et al. test the notion that laughter is an ambiguous signal, which is only interpreted correctly in the context it occurs. They provide supportive data from two experiments in which participants judged the genuineness of audio–video recordings of social interactions containing laughter (either original or replacement laughter). When replacement laughter was matched for intensity, genuineness judgments were similar to judgments of the original recordings. When replacement laughter was not matched for intensity, genuineness judgments were generally significantly lower.

Stewart et al. used the 2016 US presidential debates to study laughter together with other responses of audience, such as applause, cheering, laughter, and even booing. In three interconnected studies the impact of the norm-violating audience behavior on those watching or listening was studied. Applause–cheering significantly enhanced liking of the speaking candidate, whereas laughter did not, and party identity mediated the response to applause–cheering, but not for laughter. Thus, in such settings, cheering may be more socially contagious and laughter more stereotypic and likely to be mimicked.

The study by Auerbach confirms that it is important to distinguish between Duchenne Displays as an indicator of joy and non-Duchenne displays. Only the former go along with a variety of indicators of positive experience during a visit of hospital clowns in a rehabilitation center. Thus, also in such interventions it pays off to invest into the fine-grained assessment of facial expressions; i.e., to use the *Facial Action Coding System* to code the patients' affective responses. Only the Duchenne displays are affected by trait cheerfulness and they can serve as an indicator that hospital clown interventions are beneficial for patients.

The study of laughter also includes the dispositions to laughter—more precisely individual differences in qualities relating to laughing at and being laughed at. They are still the new kid on the block of variables related to humor and laughter with a research tradition of about 10 years. Gelotophobia (i.e., the fear of being laughed at) represents one form of humorlessness and gelotophobes see humor and laughter as weapons directed at them not as a basis for a pleasant experience to be shared with others. Together with gelotophilia (i.e., the joy of being laughed at) and katagelasticism (i.e., the joy of laughing at others) gelotophobia forms the dispositions to being laughed at and ridicule.

Two of the articles in the present collection of articles relate to their assessment. Ruch et al. utilize a picture completion task to derive a more unobtrusive semi-projective test of gelotophobia. This alternative instrument for the assessment of gelotophobia turns out to yield comparable results to the standard assessment. Hofmann et al. fulfill the need for an ultra short instrument for the assessment of these three dispositions and extends research into the workplace. They propose (and confirm in a nationally representative sample of employees) that if friendly teasing and laughter of co-workers, superiors, or customers are misperceived as malicious, one may feel less satisfied with work and life and experience more work stress. Conversely, gelotophilia went along with positive evaluations of one's life and work, and katagelasticism was negatively related to work satisfaction and positively related to work stress. Torres-Marín et al. provide evidence that gelotophobia is related to a potential bias in gaze discrimination in two experiments. Interestingly, the nature of the emotion did not play the expected role raising the question what elements are necessary for smiling faces to elicit the effect among gelotophobes.

Renner and Manthey investigate humor creation abilities in their study of self-presentation styles and dispositions to ridicule and being laughed at. They derive scores for quantitative (e.g., number of punch lines) and qualitative (e.g., wittiness of the punch lines and wittiness of the person as evaluated by three independent raters) aspects of humor creation abilities. Results show that both gelotophilia and histrionic self-presentation are supported by fluency and quality of humor creation abilities.

Three manuscripts examine gelotophobia in circumscribed groups. Kohlmann et al. investigated the associations between the experience of weight-related teasing and mockery with overweight, self-perceptions of weight, and gelotophobia in youth. Deviations from normal weight were related to experiencing teasing, which in turn was related to the fear of being laughed at. The four studies suggest that research on well-being of youth with weight problems would benefit from studying weight-related teasing and mockery in connection with gelotophobia. Tsai et al. study the relation between the dispositions toward ridicule and being laughed at, personality, and presence of autism spectrum disorder (ASD) in high school students. As in prior studies, the ASD group was found to have a higher level of gelotophobia and the present study reveals that they also have lower levels of gelotophilia and katagelasticism. However, extraversion fully accounted for the observed lower gelotophobia scores among the ASD sample, and partly for the differences found for gelotophilia. Brück et al. investigated the prevalence of gelotophobia among Borderline Personality Disorder patients. They showed an extraordinarily high level of the fear of being laughed at (i.e., 87%) compared to other clinical and non-clinical reference groups.

### Playfulness

The section on playfulness consists of five contributions of which two have a qualitative approach, while the others are quantitative in nature. Two contributions focus on *play* (the behavior associated with trait playfulness) and playfulness in school and the others employ adult samples. With 1,235 Tweets reaching an upper bound of 3,945,511 followers (March 25th, 2019)^1^, Barnett's article attracted much attention on social media. Her analyses show that teachers react differently—more negatively—toward playfulness expressed by boys than by girls (kindergarten-aged children followed up across 3 years). In contrast, playfulness in girls did not seem to be a concern for the teachers. The methodology employed and the study of gender differences provides a valuable update on earlier literature. Overall, the emerging question is how teachers, schools and societies in general may benefit from playfulness in the classroom.

Pinchover's pilot study examines the interplay of playfulness in teachers and their students. Taking the limitations of this initial study into account, this may indicate that teacher behavior impacts children's playfulness. Given that there is initial evidence for a contribution of playfulness to academic achievement and more robust data on a beneficial use for stress coping, some functions of playfulness may be helpful for students in their learning experience and development.

The idea that a playful state of mind contributes to innovativeness and creativity has received much interest in the literature (for overviews see Proyer et al., 2019) and, for example, it has been argued that “[…] a child who experiences truly “playful play” learns cognitive and behavioral processes that enhance his creative potential” (Bishop and Chace, 1971; p. 321). Heimann and Roepstorff introduce microphenomenological interviews as a method for research in playfulness. In this initial study, they found that autonomy and self-expression were of particular importance for achieving a playful state of mind.

Proyer et al. test associations of playfulness with self-reported health, activity, and physical fitness. Self- and peer-ratings (i.e., ratings by knowledgeable others; Study 1) and a series of behavioral tests (Study 2) to assess playfulness were collected. Overall, playfulness is linked to some facets of physical functioning. Future research will have to clarify the pathways and moderators of these associations (e.g., causality or indirect ways of impacting greater physical activity).

Finally, Pang and Proyer present first data on a comparison of playfulness scores in samples from two regions in the P.R. China and a sample from German-speaking countries—using measures from both, the East and the West. The article provides details on cultural differences and linguistic challenges in the translation of the term playfulness. Overall, the findings indicate that differences are smaller than expected, but that the differentiation between private and public situations impacts how people in the two regions enjoy expressing their playfulness. This study narrows a gap in the literature by providing initial data on cross-cultural differences (see also Barnett, 2017) and highlights that larger scale cross-cultural comparisons are encouraged.

These five studies support the notion that playfulness has an impact on various domains of life, but also that more research will be needed for a better understanding of its role across different age groups.

### Cheerfulness

Cheerfulness has a tradition in psychological research for more than 100 years (e.g., Morgan et al., 1919). Trait cheerfulness, seriousness, and bad mood have been proposed to form the temperamental basis of humor. Bypassing the vague folk concept of the “sense of humor” they were expected to predict humor-related thoughts, feelings, and actions. Washburn in her early studies claimed that a person in the attitude of cheerfulness is incapable of a depressing thought, and meanwhile there is ample evidence that trait cheerful individuals maintain being in a cheerful state (i.e., keep humor) in the face of adversity. The contributions of the present collection of articles are diverse. First, a training of humor yielded outcomes for cheerfulness, seriousness, and bad mood) in the desired direction with medium to large effect sizes (Tagalidou et al.). Different to a recent study (Ruch et al., 2018) the state version was utilized. Congruent with the assumption that cheerfulness predicts smiling and laughter, Auerbach shows that trait cheerful patients showed more genuine smiling and laughter during a hospital clown intervention than low trait cheerful individual do. Hofmann et al. present an adaptation of the instrument measuring state and trait cheerfulness using samples from the USA and the UK to providing the basis for studies with English-speaking participants. Next to the long version with 106 items, they provide the standard short form with 60 items and deliver initial validation data. López-Benítez et al. investigate a cognitive mechanism associated with trait cheerfulness. Utilizing a task-switching paradigm they find that while trait cheerfulness does not influence switching costs it modulates preparation and repetition effects. Studies like this are needed to further illuminate the processes associated with the traits be it cheerfulness, playfulness, or humor. Bruntsch and Ruch find trait cheerfulness and low bad mood facilitating the detection of ironic praise.

## Conclusions

The individual contributions show how humor, laughter, playfulness, and cheerfulness are related and yet heterogeneous. Each field would profit from starting to talk to each other, see overlaps in scope, finding common structure, common language, and work on theories connecting these fields. Combining the domains in the prediction of important criteria might be important too. The topics studies in this research topic (plus others) may be understood as nodes in a larger net and the interrelations need to be better explored.

It is positive to see that integrative models within the domains are now developed. This indeed needs to be the prime goal, namely to work on a solid structure within the four fields. It took research of personality and intelligence more than half a century to arrive at models that are shared by many. Also in these fields we once had “schools” that did believe into one model and defended it a lifetime. Later generations of researchers then found that the competing models were incomplete variants and do fit into a more general, often hierarchical model. We recommend concerted efforts to solve those basic questions, perhaps by compiling special issues on pertinent topics.

## Author Contributions

All authors listed have made a substantial, direct and intellectual contribution to the work, and approved it for publication.

### Conflict of Interest Statement

The authors declare that the research was conducted in the absence of any commercial or financial relationships that could be construed as a potential conflict of interest.

## Footnotes

^1^https://frontiers.altmetric.com/details/33125117/twitter
